# Hypoxia Increases Thyroid Cancer Stem Cell-Enriched Side Population

**DOI:** 10.1007/s00268-017-4331-x

**Published:** 2017-11-22

**Authors:** K. Mahkamova, N. Latar, S. Aspinall, A. Meeson

**Affiliations:** 1 Institute of Genetic Medicine, Faculty of Medical Sciences, Newcastle University, Newcastle-upon-Tyne, NE1 3BZ UK; 2 Northumbria Healthcare NHS Foundation Trust, North Tyneside General Hospital, Rake Lane, North Shields, NE29 8NH UK

## Abstract

**Introduction:**

Hypoxic stress is a feature of rapidly growing thyroid tumours. Cancer progression is thought to be driven by a small population of tumour cells possessing stem cell properties. Hypoxia-inducible factors (HIFs) are important mediators of hypoxia. Both HIF-1alpha and HIF-2alpha have been reported to be expressed in thyroid cancers. There is growing evidence that the HIF pathway plays a significant role in the maintenance of thyroid cancer stem cells (CSC).

**Methodology:**

We have isolated thyroid CSC from a papillary thyroid cancer-derived cell line (BCPAP) and an anaplastic thyroid cancer-derived cell line (SW1736) as side population (SP) cells (a putative stem cell population) and treated them with cobalt chloride (II) to induce hypoxia.

**Results and discussion:**

We observed an increase in the SP of cells within the thyroid cancer cell lines following induction of hypoxia.

## Introduction

The worldwide incidence of thyroid cancer has been on the increase over the past three decades [1]. The rising trend is attributed to a better detection of subclinical cases, though the possibility of increasing incidence due to other factors is argued by some studies. Differentiated thyroid cancers arising from the follicular epithelial cells of the thyroid gland (papillary and follicular thyroid cancers) are the most prevalent forms of thyroid cancer and have a relatively good prognosis with over 75% survival rate at 10 years [2]. Tumour recurrence and formation of distant metastases develop in 6–20% of cases and are the main cause of thyroid cancer-related deaths [2–5].

In contrast, anaplastic thyroid cancer (ATC, which also arises from the follicular epithelial cells) is an undifferentiated thyroid cancer characterised by rapid growth and high metastatic potential. ATC is often associated with pre-existing goitre or differentiated thyroid cancer [6]. Clinical management of ATC presents a significant challenge due to the limited role of surgical intervention at the time of diagnosis and limited response to other treatment modalities such as radiotherapy and chemotherapy, which leads to patients’ demise within 3–7 months of the diagnosis [6]. As disease advances, thyroid tumours may be subjected to hypoxic stress which results in activation of hypoxia signalling pathways, involving hypoxia-inducible factors (HIFs). HIFs are composed of an oxygen-sensitive alpha subunit, which is degraded under normoxic conditions and a stable beta subunit. HIF complex formed under hypoxic conditions acts as transcriptional factor for multiple gene targets, mainly involving cell cycle regulation (e.g. MYC, p53), anaerobic metabolism (Glut 1), intracellular pH maintenance (CA9, also known as CAIX) and angiogenesis (e.g. VEGF) allowing tumour cells to quickly adapt to the hostile environment [7]. Growing evidence shows that overexpression of HIF-1alpha and HIF-2alpha in thyroid cancers is associated with more advanced tumour grade and the presence of metastasis [8, 9]. Although HIF pathways appear to play a significant role in thyroid cancer progression, the exact mechanism remains unknown.

Histologically, thyroid tumours comprise heterogeneous populations of tumour cells, including a small population of undifferentiated cells that exhibit stem cell properties, such as unlimited proliferative potential, clonogenicity, capacity for asymmetric division and advanced cell repair mechanisms as well as the presence of efflux pumps. Cancer stem cells (CSC) have been previously identified in other types of solid tumours, with evidence, suggesting that the hypoxic microenvironment promotes the undifferentiated state of the CSC [10, 11]. Treatment modalities such as radioiodine therapy, radiotherapy and chemotherapy target metabolically active rapidly dividing mature thyroid cancer cells, but not CSC, which survive and can give rise to new thyroid tumours.

Thyroid CSC have been previously isolated based on the presence of cell surface markers, including CD133 and CD44, their potential to generate thyrospheres, the presence of putative stem cells markers such as Oct4, Sox2 and Nanog, fluorescent-activated cell sorting (FACS) based on ALDH activity and as side population (SP) cells. However, the use of surface markers to identify thyroid CSC has been controversial as many studies demonstrated that these are not uniquely or consistently expressed by thyroid CSC.

The SP assay has been used to successfully isolate thyroid stem cell-enriched SP from thyroid tissue and thyroid cell lines [12–15]. The SP analysis is based on the cell’s ability to efflux vital dyes most commonly Hoechst 33342 dye, with this efflux property being mediated by ABC-transporters, and this can be reversed using ABC-transporter inhibitors such as verapamil.

In this study, we assess the effect of hypoxia on the SP fraction in thyroid cell lines representing normal follicular thyroid cells, papillary thyroid cancer and anaplastic thyroid cancer.

## Methodology

### Cell lines

Nthy ori 3-1 representing human follicular thyroid cells were obtained from the European Collection of Cell Cultures (ECACC). BCPAP (PTC-derived) and SW1736 (ATC-derived) cell lines were kindly provided by Professor G. Brabant (Lubeck, Germany) and Professor McCabe (Birmingham, UK). BCPAP and SW1736 harbour a BRAF V600 E mutation which is present in estimated 45% PCT and 20% of ATC [16, 17]. All cell lines were verified using STR fingerprinting. Nthy ori 3-1 and BCPAP cells were cultured in RPMI 1640 culture medium, and SW1736 was cultured in high-glucose DMEM medium supplemented with 2 mM l-glutamine (Sigma), 10% foetal bovine serum (Life technologies) and 100 Iu/ml penicillin and streptomycin (Gibco).

### Cobalt chloride (II) hexahydrate (CoCl_2_) treatment

Cobalt chloride (a hypoxia-mimetic) was used to induce hypoxia in thyroid cell lines. The cells were treated with 100 µM for 48 h. Untreated cells were used as controls. Activation of HIF pathway was confirmed by PCR on the treated cells. Results were compared to thyroid cells incubated at 0.5% O_2_ for 48 h in a Ruskinn hypoxia chamber.

### Semi-quantitative PCR

RNA extraction was performed using RNAeasy Plus Micro Kit (Qiagen) as per manufacturer’s instructions. RNA concentration was measured, and RNA integrity was checked using gel electrophoresis on a 2% agarose gel. cDNA was made with Tetro cDNA synthesis kit (Bioline) using equal amount of RNA for all samples and as per manufacturer’s instructions. PCR was performed using 12.5 μl PCR mastermix (Promega), 0.5 μg/μl primers (Life technologies), 2.5 µl cDNA template and nuclease free water, to make up a final reaction volume of 25 µl. Primer sequences used are provided in Table 1. PCR cycle programme was as follows: denaturation 30 s at 94 °C, annealing temperature optimised for each primer for 30 s, elongation 1 min at 72 °C, 35 cycles. The amplified products were run on a 2% agarose gel and visualised under UV light using a GelDoc-It imager (UVP). Band intensity was measured using image J programme. GAPDH was used for standardisation.

**Table 1:** Sequence of primers used in standard PCR

Gene	Primer sequence	Product length (base pairs)
Oct3/4	Forward: AGTGAGAGGCAACCTGGAGA Reverse: ACACTCGGACCACATCCTTC	110
Sox2	Forward: AACCCCAAGATGCACAACTC Reverse: GCTTAGCCTCGTCGATGAAC	100
Nanog	Forward: TTCCTTCCTCCATGGATCTG Reverse: TCTGCTGGAGGCTGAGGTAT	213
HIF-1alpha	Forward: TCATCCAAGAAGCCCTAACG Reverse: TCGCTTTCTCTGAGCATTCTG	112
HIF-2alpha	Forward: TCTGAAAACGAGTCCGAAGCC Reverse: GGTCGCAGGGATGAGTGAAGT	196
CA9	Forward: ATCGCTGAGGAAGGCTCAGA Reverse: AGGGTGTCAGAGAGGGTGTG	195
VEGF	Forward: TGTGTGTGTGTGAGTGGTTGA Reverse: TCTCTGTGCCTCGGGAAG	69
GAPDH	Forward: GCACCGTCAAGGCTGAGAAC Reverse: GCCTTCTCCATGGTGGTGAA	150

### Side population assay

Cells were incubated in low-serum media (2%FBS) and Hoechst 33342 dye using a pre-optimised SP protocol for each thyroid cell line. 100 µM of verapamil (Sigma) was added to block ABC-transporters efflux ability. Post-incubation, the cells were washed with ice-cold PBS, filtered and analysed on LSR Fortessa flow cytometer (BD Biosciences). Cell debris and non-viable cells were identified using propidium iodide and excluded from the analysis as well as doublet cells. Data were analysed using BD FACSDiva software (version 8).

## Results

### Expression of hypoxia markers

**Fig. 1:**
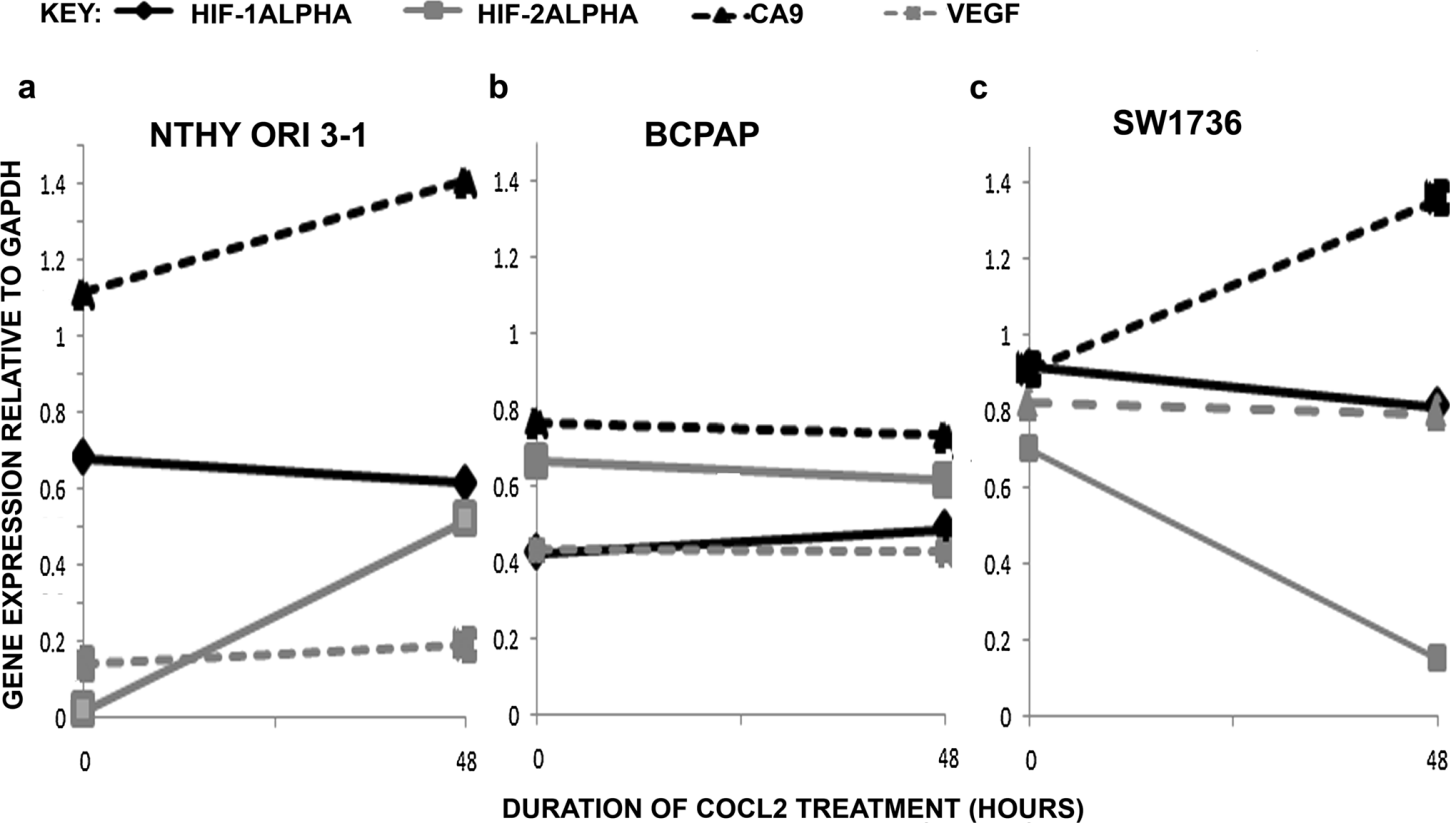
Graphs showing relative mRNA expression of the hypoxia markers in the untreated sample and after 48 h of treatment with cobalt chloride in Nthy ori 3-1 (**a**), BCPAP (**b**) and SW1736 (**c**). *n* = 1

Standard PCR was performed to confirm HIF pathway activation by measuring mRNA expression of HIF-1alpha, HIF-2alpha and their downstream gene targets CA9 and VEGF in the thyroid cell lines treated with 100 µM of COCl_2_ for 48 h (Fig. 1).

HIF-1alpha mRNA was strongly expressed in all three untreated samples. Following hypoxia induction, the levels remained relatively similar in all three treated samples compared to the controls.

HIF-2alpha mRNA was expressed in the untreated BCPAP and SW1736, but not in Nthy ori 3-1. Following CoCl_2_ treatment, the mRNA expression sharply rose in Nthy ori 3-1, but a downward trend in expression was observed in BCPAP and SW1736.

The mRNA levels of HIF-1alpha and HIF-2alpha, CA9 and VEGF increased in Nthy ori-3-1 and SW1736 following induction of hypoxia, but remained unchanged in the treated BCPAP.

### The effect of hypoxia on SP population

**Fig. 2:**
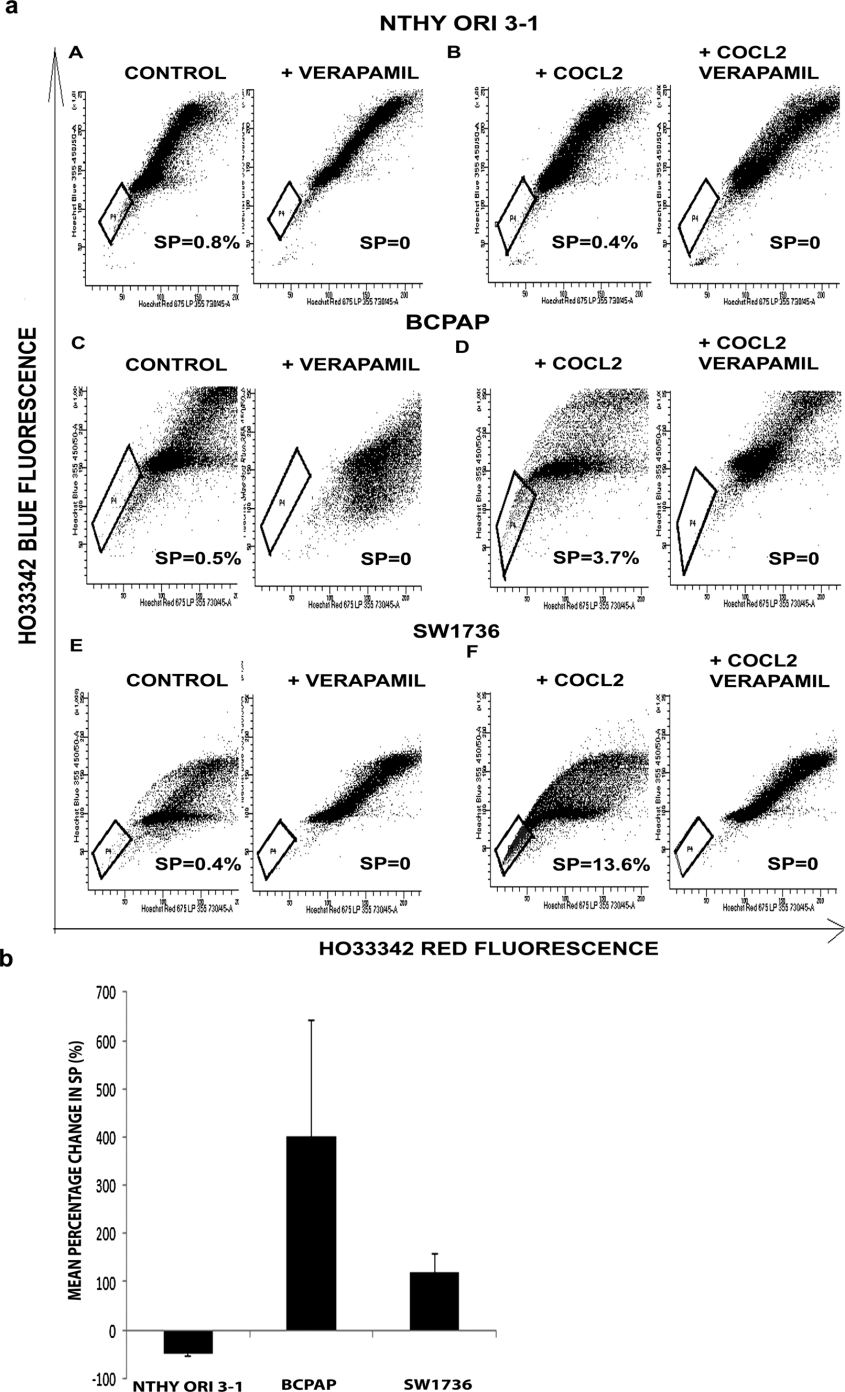
**a** FACS plots showing SP in Nthy ori 3-1 before (A) and after treatment with 100 μM of cobalt chloride (B), in BCPAP before (C) and after treatment with 100 μM of cobalt chloride (D), and in SW1736 before (E) and after treatment with 100 μM of cobalt chloride (F). In each case, SP phenotype was confirmed by addition of verapamil. Note decrease in SP in Nthy ori 3-1 and increase in SP in BCPAP and SW1736 following induction of hypoxia. **b** Graphical representation of flow cytometry data from three independent experiments of the SP analysis following cobalt chloride-induced hypoxia. Results are expressed as mean percentage change in SP, and bars represent SEM (*p* = 0.09 Nthy ori 3-1, *p* = 0.344 BCPAP, *p* = 0.404 SW1736)

SP analysis was performed on the three cell lines cells treated with 100 µM of CoCl_2_ for 48 h. Untreated cells were used as control (Fig. 2).

The untreated Nthy ori 3-1 (A) BCPAP (C) and SW1736 (E) all contained a small SP cell population. However, following hypoxia induction with CoCl_2_, the Nthy ori 3-1 SP fraction was reduced (B), while there was a significant increment in SP fractions in both the BCPAP (D) and SW1736 (F) cells. A graphical representation of data from three independent experiments of the SP analysis following hypoxia induction showing the mean percentage change in SP for each cell line (2b) confirms the impact of hypoxia treatment, with mean SP reduction of 68% for Nthy ori 3-1, and mean SP increase of 400% for BCPAP and 120% for SW1736, however, changes for all 3 where not significant using a two-sided t test.

### The effect of hypoxia on stem cell marker expression

**Fig. 3:**
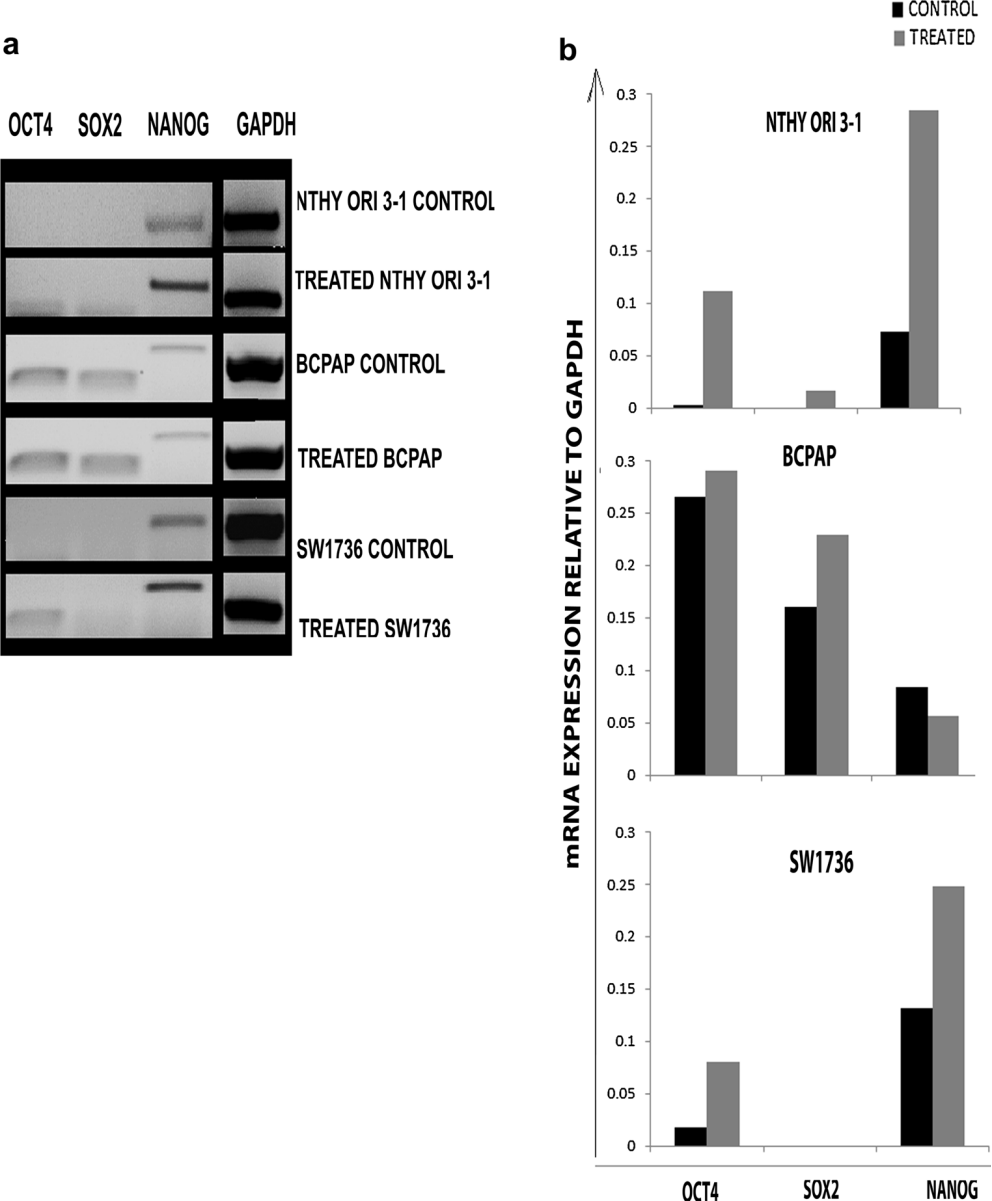
**a** PCR products of stem cells markers gene expression in untreated Nthy ori 3-1, BCPAP and SW1736 and following treatment with cobalt chloride. GAPDH was used as housekeeping gene. **b** Graphs showing change in the expression of mRNA of putative stem cell markers following hypoxic treatment. The values shown are relative to housekeeping gene GAPDH

Transcription factors Oct4, Sox2 and Nanog regulate expression of genes involved in maintaining pluripotency and regulation of self-renewal. Using standard PCR, mRNA expression was examined in untreated and CoCl_2_-treated cells (Fig. 3).

Following hypoxia treatment, the levels of Oct4 and Nanog mRNA increased in Nthy ori 3-1 and Sox2 mRNA levels became detectable in the treated sample. In BCPAP, hypoxia resulted in upregulation of Sox2 and Oct4 with slight reduction in Nanog mRNA levels. No Sox2 expression was detected in the treated or untreated SW1736, but stronger expression of Oct4 and Nanog was seen following hypoxic treatment.

## Discussion

The CSC model for thyroid carcinogenesis, which is based on the principle that a small subset of the cells within tumour bulk has the capacity to generate and propagate tumours, has become more widely acceptable in the recent years due to identification of thyroid cancer cell populations possessing a stem cell phenotype. Longer life span and aberrant cell cycling regulation mean that CSC are more likely to accumulate genetic mutations necessary to transform into cancerous cells unlike normal tissue cells [10]. Thyroid CSC origin remains debatable with speculations that CSC may be transformed stem cells residing in the thyroid tissue or that they may have arisen as a result of dedifferentiation of the mature thyroid cancer cells.

In our experiments, we used cobalt chloride to induce a hypoxic state in thyroid CSC. Cobalt chloride acts by reducing intracellular ascorbate levels which promotes iron oxidation. Iron acts as a substrate for the enzyme propyl hydroxylase, which is responsible for hydroxylation of HIF-alpha subunits. This stops binding of von Hippel–Lindau protein and E3 uniquitin ligase to HIF-alpha and results in its subsequent degradation [18]. This method of inducing hypoxia has been used in a number of studies [9, 19].

The SP assay is a validated technique that was used to isolate CSC-enriched population from different types of solid organ tumours including breast, gastric, intestine, prostate and thyroid [12, 20]. We previously determined that sorted SP from SW1736 and BCPAP exhibits stem cell characteristics such as their ability to generate both SP and non-SP (asymmetric division) and had higher expression of the stemness markers and lacked expression of thyroid differentiation markers (data not shown). All three thyroid cell lines used in the study were found to contain an SP. Although the SP fraction was consistently lower in the normal thyroid cell line Nthy ori 3-1(around 0.1–0.5%), SP value in the two thyroid cancer cell lines was found not to be related to the thyroid tumour type.

Previously, both HIF-1alpha and HIF-2alpha were found to be expressed at protein level in thyroid cancer tissues, but not in the normal tissues and their overexpression was found to be correlated with tumour aggressiveness and metastases [21, 22].

In our study, we observed differences in HIFs mRNA expression in the treated cell lines, suggesting that the hypoxia may differently regulate HIF-1alpha and HIF-2alpha expression in normal and cancerous thyroid cell lines, thus leading to activation of different cellular pathways. HIF-1alpha was found to be expressed in all three thyroid cell lines during normoxia, and the levels remain relatively stable after 48 h of hypoxic treatment which may be reflective of the fact that post-transcriptional and post-translational regulation of HIF-1alpha occurs, and measurement of protein levels rather than mRNA levels would be more accurate.

HIF-2alpha was upregulated in the normal thyroid cell line following hypoxia treatment. When Nthy ori 3-1 was treated with CoCl_2_ for 2, 24, 48 and 72 h, HIF-1alpha levels increased on induction of hypoxia and then declined over time and the opposite pattern was seen with HIF-2alpha expression (data not shown), suggesting that HIF-1alpha may mediate acute phase response to hypoxia and HIF-2alpha target genes may be recruited in the chronic hypoxic response. HIF-2alpha can mediate stem cell phenotypes by directly inducting Oct4 transcription, and in our experiments we observed increased stem cell markers expression following CoCl_2_ treatment, but a fall in the SP fraction in the treated Nthy ori 3-1 after 48 h, possibly due to the normal thyroid cells being more sensitive to the effects of hypoxic treatment with more cells succumbing to hypoxia than proliferating.

The presence of BRAF mutation in BCPAP and SW1736 can aberrantly activate HIF pathway independent of hypoxia, promoting overactive MEK/EPK signalling which results in increased HIF protein synthesis, suggesting that activation of the HIF pathway is essential in thyroid cancer progression [8]. This is supported by our finding that HIF-2alpha mRNA was detected only in the cancerous thyroid cell lines in the normoxic state and the levels remained relatively unchanged following hypoxia induction. In SW1736, both HIF-1alpha and HIF-2alpha mRNA expression decreased with CoCl_2_ treatment; however, the target genes CA9 and VEGF were upregulated, confirming that the pathway had been activated. In the BCPAP cell line, we did observe mRNA for CA9 remained unchanged even after hypoxia treatment. This may be a limitation of the analysis method used as changes may have been too small to detect or it may be that mRNA levels were unaltered, but changes in protein expression might still occur. Burrows and colleagues did demonstrate an increase in CA9 in the BCPAP cell line following induction of hypoxia using cobalt chloride (II) at the protein level [9].

In both thyroid cancer cell lines, hypoxia resulted in an increase in the SP fraction and upregulation of stem cell markers mRNA; however, further experiments would be required to confirm statistical significance of these findings. Both HIF-1alpha and HIF-2alpha can induce CSC populations, but appear to do so by activating different stem cell pathways. HIF-1alpha is responsible for stabilising Notch 1, another important pathway in promoting undifferentiated state of the cells, as well as promoting invasive and metastatic characteristics of the tumour by activating transcription factors regulating epithelial-to-mesenchymal transition (EMT), one of the main processes by which metastases are thought to occur. Furthermore, cells undergoing EMT can generate tumour cells with cancer stem cell characteristics [23].

Although it had been previously described that hypoxia markers were related to the higher degree of thyroid tumour dedifferentiation and invasiveness, this is the first study that looked at the association between hypoxia, HIFs and thyroid cancer stem cells.

It’s becoming increasingly evident that eradication of solid organ tumours would require elimination of the cancer stem cell population, in which the current treatment modalities such as chemotherapy, radiotherapy and radioactive iodine ablation (RAI) fail to target. Targeting HIF activity could ultimately lead to reduction in thyroid CSC numbers, thus impairing regeneration and metastatic potential of thyroid tumours and making them more susceptible to the effects of RAI, radiotherapy and chemotherapy. There are a number of agents in clinical trials that target HIF’s activity as well as inhibiting the members of the HIF signalling pathways (Table 2). Furthermore, CSC markers have been shown to be reliable predictors of tumour recurrence in other types of solid tumours and their role in disease surveillance in thyroid cancers should be further explored [24].

**Table 2:** Summary of most recent clinical trials targeting HIFs and their downstream target genes

Drug name	Mechanism of action	Neoplasm	Clinical trial phase	Clinical trial identification number
EZN-2968	HIF-1alpha mRNA antagonist	Liver, lymphoma	1	NCT 0176393
PT2385	HIF-2alpha inhibitor	Glioblastoma	1	NCT03216499
Digoxin	HIF-1alpha inhibitor	Breast cancer	1	NCT0176393
R07070179	HIF-alpha mRNA antagonist	Liver	1	NCT02564614
PX-478	HIF-1alpha inhibitor	lymphoma	1	NCT00522652
PT2977	HIF-2alpha inhibitor	Kidney	1	NCT02293980
BAY87-2243	HIF-1alpha protein suppression	Various advanced solid organ tumours	1	NCT01297530
Bevacizumab	Anti-VEGF monoclonal antibody	Breast	3	NCT00109239
		Pancreas	1	NCT00447710
		Rectal	2	NCT00113230
Sevacizumab	Anti-VEGF monoclonal antibody	Colorectal	1	NCT02453464
Lucitanib	VEGFR/FGFR inhibitor	Lung	2	NTC02109016
Aflibercept	VEGF inhibitor	Ovarian	1	NCT00327171
		Breast	2	NCT00436501
		colorectal	2	Multiple
DTP348	CA9 inhibitor	Head and neck	1	NTC02216669

## Conclusion

Hypoxia may be contributing to progression of thyroid cancer by promoting generation of treatment-resistant stem cell-enriched SP. Two HIF isoforms, HIF-1alpha and HIF-2alpha, may play different roles in thyroid carcinogenesis. Further understanding of the cellular pathways involved is essential in the development of future treatments that could target thyroid CSC.

## References

[1] Kitahara C, Sosa J (2016). The changing incidence of thyroid cancer. Nat Rev Endocrinol.

[2] Madani A, Tabah R, How J (2015). Rare metastases of well-differentiated thyroid cancers: a systematic review. Ann Surg Oncol.

[3] Benbassat C, Hirsch D (2006). Clinicopathological characteristics and long-term outcome in patients with distant metastases from differentiated thyroid cancer. World J Surg.

[4] Durante C, Baudin E, Leboulleux S (2006). Long-term outcome of 444 patients with distant metastases from papillary and follicular thyroid carcinoma: benefits and limits of radioiodine therapy. J Clin Endocrinol Metab.

[5] Lee J (2010). Differentiated thyroid carcinoma presenting with distant metastasis at initial diagnosis clinical outcomes and prognostic factors. Ann Surg.

[6] Chiacchio S, Lorenzoni A, Boni G (2008). Anaplastic thyroid cancer: prevalence, diagnosis and treatment. Minerva Endocrinol.

[7] Majmundar AJ, Wong WJ, Simon MC (2010). Hypoxia-inducible factors and the response to hypoxic stress. Mol Cell.

[8] Burrows N, Babur M, Resch J (2011). Hypoxia-inducible factor in thyroid carcinoma. J Thyroid Res.

[9] Burrows N, Resch J, Cowen RL (2010). Expression of hypoxia-inducible factor 1 alpha in thyroid carcinomas. Endocr Relat Cancer.

[10] Moore N, Lyle S (2011). Quiescent, slow-cycling stem cell populations in cancer: a review of evidence and discussion of significance. J Oncol.

[11] Seidel S, Garvalov BK, Wirta V (2010). A hypoxic niche regulates glioblastoma stem cells through hypoxia inducible factor 2a. Brain.

[12] Mitsutake N, Iwao A, Nagai K (2007). Characterization of side population in thyroid cancer cell lines: cancer stem-like cells are enriched partly but not exclusively. Endocrinology.

[13] Lan L, Cui D, Nowka K (2007). Stem cells derived from goiters in adults form spheres in response to intense growth stimulation and require thyrotropin for differentiation into thyrocytes. J Clin Endocrinol Metab.

[14] Chen G, Xu S, Renko K (2012). Metformin inhibits growth of thyroid carcinoma cells, suppresses self-renewal of derived cancer stem cells and potentiates the effect of chemotherapeutic agents. J Clin Endocrinol Metab.

[15] Zheng X, Cui D, Xu S (2010). Doxorubicin fails to eradicate cancer stem cells derived from anaplastic thyroid carcinoma cells: characterisation of resistant cells. Int J Oncol.

[16] Xing M (2005). BRAF mutation in thyroid cancer. Endocr Relat Cancer.

[17] Guerra A, Di Crescenzo V, Garzi A (2013). Genetic mutations in the treatment of anaplastic thyroid cancer: a systematic review. BMC Surg.

[18] Salnikow K, Donald SP, Bruick RK (2004). Depletion of intracellular ascorbate by the carcinogenic metals nickel and cobalt results in the induction of hypoxic stress. J Biol Chem.

[19] Cho J, Kim D, Lee S (2005). Cobalt chloride-induced estrogen receptor α down-regulation involves hypoxia-inducible factor-1α in MCF-7 human breast cancer cells. Mol Endocrinol.

[20] Patrawala L, Schneider-Broussard R, Zhou J (2005). Side population is enriched in tumorigenic, stem-like cancer cells, whereas ABCG2+ and ABCG2− cancer cells are similarly tumorigenic. Can Res.

[21] Koperek O, Akin E, Asari R (2013). Expression of hypoxia inducible factor 1 alpha in papillary thyroid carcinoma is associated with desmoplastic stromal reaction and lymph node metastases. Virchows Arch.

[22] Wang N, Dong CR, Jiang R (2014). Overexpression of HIF-1alpha, metallothionein and SLUG is associated with high TNM stage, lymph node metastases in papillary thyroid carcinoma. Int J Clin Exp Patholog.

[23] Lan L, Cui D (2013). Epithelial-mesenchymal transition triggers cancer stem cell generation in human thyroid cancer cells. Int J Oncol.

[24] de Jong MC, Pramana J, van der Wal JE (2010). CD44 expression predicts local recurrence after radiotherapy in larynx cancer. Clin Cancer Res.

